# Tick-borne lymphadenopathy (TIBOLA) acquired in Southwestern Germany

**DOI:** 10.1186/1471-2334-11-167

**Published:** 2011-06-10

**Authors:** Siegbert Rieg, Sabine Schmoldt, Christian Theilacker, Katja de With, Silke Wölfel, Winfried V Kern, Gerhard Dobler

**Affiliations:** 1 Center for Infectious Diseases and Travel Medicine, University Hospital Freiburg, Hugstetter Strasse 55, 79106 Freiburg, Germany; 2 Bundeswehr Institute of Microbiology, Neuherbergstrasse 11, 80937 Munich, Germany

## Abstract

**Background:**

Tick-borne lymphadenopathy (TIBOLA) was first described in 1997 in a patient in France. The causative agent, *Rickettsia slovaca*, is transmitted by *Dermacentor *ticks.

**Case presentation:**

In southwestern Germany we encountered a patient with a tick bite at the dorsal scalp that resulted in an eschar and nuchal lymphadenopathy. Additionally, fever, malaise as well as elevated inflammatory markers and transaminases occurred. The characteristic clinical picture along with positive antibody testing for rickettsiae of the tick-borne spotted fever group strongly suggest the diagnosis TIBOLA.

**Conclusion:**

Human rickettsioses are emerging infections. Clinicians should be aware of TIBOLA as a newly described rickettsial disease. As in our case, TIBOLA may be encountered in regions/countries where *R. slovaca *and *Dermacentor *ticks are prevalent but autochthonous acquisition was not described before.

## Background

Rickettsiae are obligate intracellular gram negative bacteria transmitted in the natural environment by vectors such as ticks, fleas, lice, and mites. Of medical importance are Rickettsiae of the typhus group (*R. prowazekii*, *R. typhi*) and Rickettsiae of the tick-borne spotted fever group (e.g. *R. rickettsii*, *R. conorii*, *R. africae*), which now compromises more than 20 different species as several new species have been identified in recent years [1].

Tick-borne lymphadenopathy (TIBOLA) was first described in 1997 in a female patient in France and is defined as the association of a tick bite resulting in an inoculation eschar on the scalp and enlargened cervical lymphnodes in the absence of a rash [2]. Although the causative agent of TIBOLA, *Rickettsia slovaca*, is prevalent in *Dermacentor *ticks in Germany, until 2009 no case of TIBOLA has been described. Here we report the first case of TIBOLA acquired in Germany.

## Case presentation

**Figure 1:**
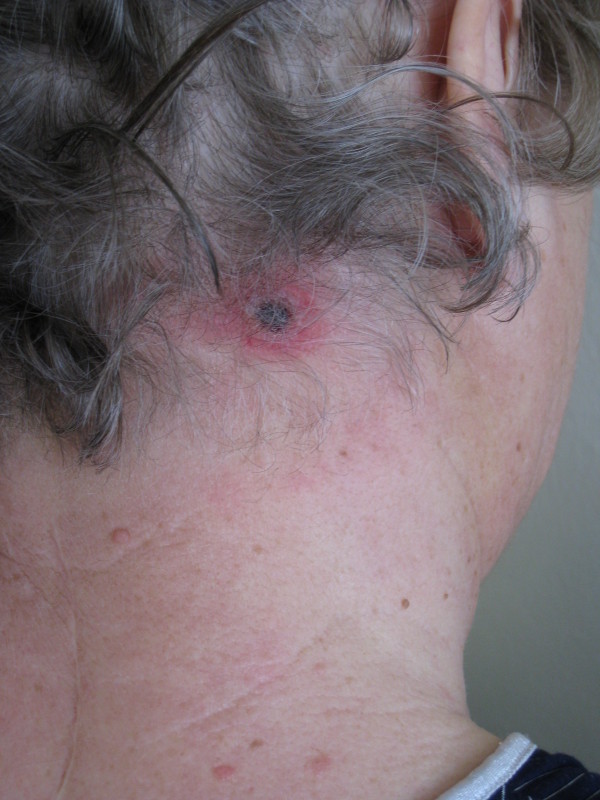
**Inoculation eschar**. A 67-year old woman with an inoculation eschar with central necrosis, edematous margins and erythematous halo at the former site of the tick bite.

**Figure 2:**
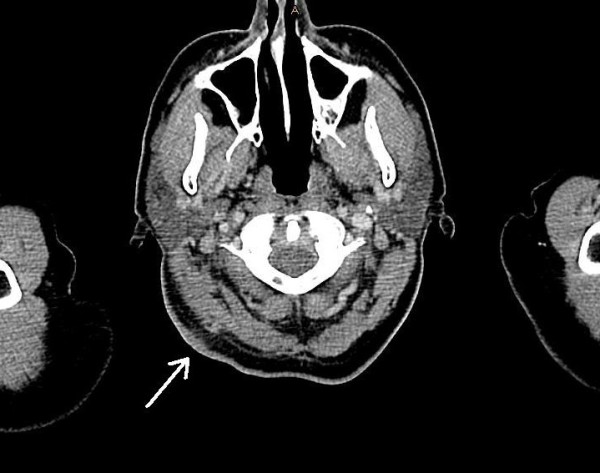
**Head computed tomography scan**. Imbibition of subcutaneous tissue underneath the eschar (arrow).

**Figure 3:**
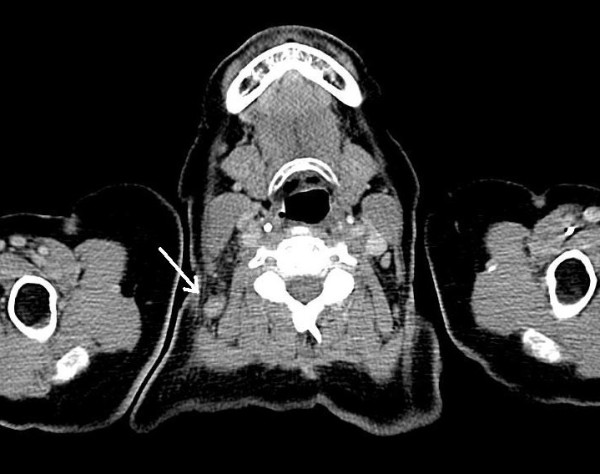
**Neck computed tomography scan**. Non-necrotizing lymphadenitis (arrow) along the draining lymph nodes without evidence of cervical or mediastinal abscess formation (courtesy of Mathias Langer, University Hospital Freiburg, Germany).

In February 2009, a 67-year-old female patient sought medical advice one week after suffering from a tick bite at the dorsal scalp. She had acquired the tick in southern Germany in the vicinity of Freiburg (Baden-Wuerttemberg). Two days after removal of the tick she noticed redness followed by ulcer formation at the biting site along with swollen regional lymph nodes. She presented with high fever (40°C), malaise, headache and nuchal lymphadenopathy. At the site of the tick bite an eschar with edematous margins was present (Figure 1). The remaining physical examination was unremarkable. She denied animal contacts and had not been anywhere else than in the vicinity of Freiburg. Laboratory investigations revealed a white blood cell count of 8.3 × 10^9^/L, C-reactive protein of 112 mg/L (normal < 5 mg/L), moderately elevated transaminases (ALT 133 U/L [normal < 35 U/L], AST 99 U/L [< 35 U/L], γ-GT 158 U/L [< 40 U/L]) and mildly elevated LDH (314 U/L [< 214 U/L]). Blood cultures were negative and cultures of dermal swabs obtained from the eschar revealed normal skin flora. Computed tomography revealed imbibition of the subcutaneous tissue underneath the eschar (Figure 2) and non-necrotizing lymphadenitis of the draining lymph nodes without evidence of cervical or mediastinal abscess formation (Figure 3). Antibody testing for *Francisella tularensis*, *Borrelia burgdorferi*, *Rickettsia prowazekii*, *Rickettsia typhi *and *Yersinia enterocolitica *was negative. She was empirically treated with ampicillin/sulbactam and ciprofloxacin, three days after initiation of antibiotic treatment defervescence and clinical improvement could be noticed.

**Figure 4:**
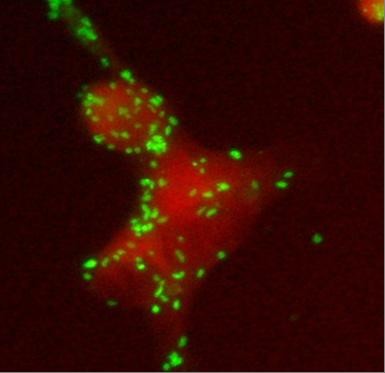
**Indirect immunofluorescence assay using *R. conorii *(dilution 1:256)**.

In serial serum samples (day 7, 17 and 24) specific antibody testing for rickettsiae of the tick-borne spotted fever group revealed a seroconversion (indirect immunofluorescence assay (Figure 4): *R. conorii *IgG [Fuller Laboratories], <1:32, 1:512, 1:1024, respectively; *R. helvetica *IgG [in house], <1:32, 1:32, 1:128, respectively). Skin-biopsy of the eschar was not performed. The patient recovered without further complications, a change to specific anti-rickettsial antibiotic therapy, i.e. doxycylin was not necessary.

**Figure 5:**
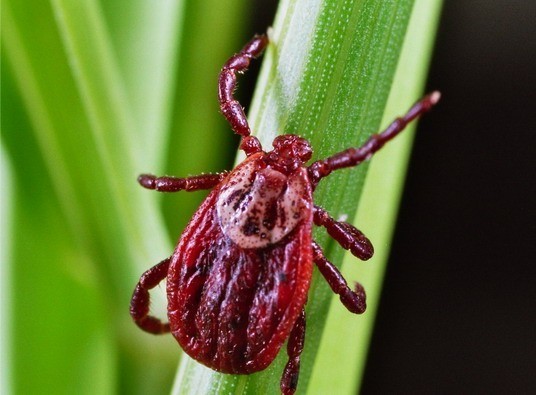
**Adult *Dermacentor *spp**. ticks are vectors of *R. slovaca *and *R. raoultii*. *D. marginatus *and *D. reticulatus *tick bites are preferentially located in the scalp region, the majority of tick bites occur during the colder months. *R. slovaca *and *R. raoultii *are maintained within *Dermacentor *spp. by transovarial and transstadial transmission (courtesy of Heinz Mehlhorn, Duesseldorf University, Germany).

Human rickettsioses occur worldwide and are considered to be emerging infections [3]. One of the newly discovered rickettsial diseases is tick-borne lymphadenopathy (TIBOLA). The causative agent, *Rickettsia slovaca*, was discovered in 1968 in Slovakia, but was being considered as non-pathogenic until 1997 [2,4]. *R. slovaca *is transmitted almost exclusively by *Dermacentor marginatus *and rarely by *Dermacentor reticulatus *ticks (Figure 5).

Here we report the case of a female Caucasian patient presenting with the characteristic clinical features of TIBOLA. In our patient the tick was not available for genus and species identification, however, the season late winter/early spring and the localisation of the tick bite on the scalp strongly suggest a bite due to *D. marginatus *which is prevalent in the vicinity of Freiburg. Noteworthy, TIBOLA is one of the rare tick-borne diseases that is more common in the cold season. As reported in several case series, females are at higher risk of TIBOLA, the mean age is 30-35 years with a substantial proportion of cases occurring in children. Apart from TIBOLA, two other acronyms were proposed for this clinical entity: *Dermacentor*-borne necrosis erythema and lymphadenopathy (DEBONEL) or scalp eschar and neck lymphadenopathy after tick bite (SENLAT) [5].

*R. slovaca *has been described in Dermacentor ticks in many European countries including France, Greece, Hungary, Spain, Italy, Switzerland, Austria, Russia, Ukraine, Armenia, and others. In Germany, *R. slovaca *has been found in *D. marginatus *ticks in Baden-Wuerttemberg in the area of the Rhine valley [6] and recently in the Main valley in the vicinity of Aschaffenburg, Bavaria [7].

Within the last years, detection of *R. slovaca *from human cases has been increasingly reported from France, Hungary, Spain and Italy [4,8-10]. It is remarkable that although *R. slovaca *was isolated in ticks in Germany already 30 years ago, no human case of TIBOLA in Germany has been described until recently. As a consequence of the case presented here and another case of 2009 from southern Rhineland-Palatinate [7], Germany must be added to the list of countries where autochthonous acquisition of TIBOLA has occurred. *R. slovaca *infections seem to be more widespread over Germany as has been found so far since our case was several hundred kilometers south of the case described in Rhineland-Palatinate. Including the recently described detection of *Rickettsia aeschlimannii*, up to now seven rickettsial species have been reported in Germany [11].

Apart from *R. slovaca*, the rickettsial genotypes RpA4, DnS14 and DnS28 belonging to a new spotted fever group species recently named *R. raoultii *have been implicated in the etiology of TIBOLA [12]. Due to only minor antigenetic differences among all spotted fever group rickettsiae, serology is not able to discriminate within members of this group and unambiguous (sub)species-specific diagnosis can only be made by PCR from skin biopsies or swab specimen of the eschar [13]. As *R. raoultii *is prevalent in *D. reticulatis *ticks in southern Germany and skin biopsy/PCR was not performed, we cannot rule out TIBOLA due to *R. raoultii*. However, our patient suffered from high fever and pronounced malaise. This strongly favours an infection due to *R. slovaca *since *R. raoultii *is considered to be less pathogenic and to cause a milder form of TIBOLA [12,14].

Noteworthy, apart from fever and malaise our patient presented with moderately elevated transaminases indicating hepatic involvement in the course of infection. Further evaluation included abdominal/hepatic ultrasonography which was unremarkable. Antibody testing for *Coxiella burnetii *was negative. Improvement of ALT/AST during antibiotic treatment argues against drug induced hepatitis due to ampicillin/sulbactam or ciprofloxacin.

The differential diagnosis in patients with an eschar includes rickettsial infections, cutaneous anthrax, tularaemia, necrotic arachnidism (brown recluse spider bite), scrub typhus (*Orientia tsutsugamushi*), rat bite fever (*Spirillum minus*), staphylococcal or streptococcal ecthyma, and, as discussed recently, infection due to *Bartonella henselae *[5]. However, in the context of a previous tick bite, infection due to *Francisella tularensis*, or possibly *B. henselae*, as well as staphylococcal or streptococcal superinfection occur as most likely diagnoses.

As for other rickettsioses the treatment of choice is considered to be a 7 to 10 day course of doxycycline (100 mg twice daily for adults), with ciprofloxacin or azithromycin/clarithromycin as possible alternative agents. Severe complications as seen in other rickettsiosis (e.g. epidemic typhus, Rocky Mountain Spotted Fever, Mediterranean Spotted Fever) have not been described in TIBOLA so far.

## Conclusions

Our knowledge on geographic distribution, epidemiology and ecology of rickettsiae is currently evolving. The prevalence of rickettsial diseases in Germany and elsewhere is probably underestimated. TIBOLA should be noticed as an emerging infection, partly due to an increasing spread of *Dermacentor marginatus *ticks. Clinicians should be aware of TIBOLA as a tick-borne disease as well as of the local prevalence of rickettsial species and their potential vectors. In typical cases (as in the patient reported here) awareness of TIBOLA enables a rather reliable clinical diagnosis that typically results in incisive diagnostic approaches and simple, unexpensive treatment.

## Consent

Written informed consent was obtained from the patient for publication of this case report.

## Competing interests

The authors declare that they have no competing interests.

## Authors' contributions

SR took care of the patient and drafted and wrote the manuscript. SSch confirmed immunofluorescence assays and contributed to draft the manuscript. CT took care of the patient and revised the manuscript. KdW took care of the patient and revised the manuscript. SW performed and interpreted immunofluorescence assays and contributed to draft the manuscript. WVK coordinated and edited the manuscript. GD confirmed immunofluorescence assay results and contributed in coordination and editing of the manuscript. All authors have read the manuscript and approved its final version.

## Pre-publication history

The pre-publication history for this paper can be accessed here:

http://www.biomedcentral.com/1471-2334/11/167/prepub
